# Restoration of FVIII expression by targeted gene insertion in the *FVIII* locus in hemophilia A patient-derived iPSCs

**DOI:** 10.1038/s12276-019-0243-1

**Published:** 2019-04-17

**Authors:** Jin Jea Sung, Chul-Yong Park, Joong Woo Leem, Myung Soo Cho, Dong-Wook Kim

**Affiliations:** 1 Department of Physiology, Yonsei University College of Medicine, 50-1 Yonsei-ro Seodaemun-gu, Seoul, 03722 Korea; 2 Brain Korea 21 PLUS Program for Medical Science, Yonsei University College of Medicine, 50-1 Yonsei-ro, Seodaemun-gu, Seoul, 03722 Korea; 3 Severance Biomedical Research Institute, Yonsei University College of Medicine, 50-1 Yonsei-ro Seodaemun-gu, Seoul, 03722 Korea; 4 S. Biomedics Co., Ltd, Seoul, South Korea

**Keywords:** Genetic engineering, Induced pluripotent stem cells

## Abstract

Target-specific genome editing, using engineered nucleases zinc finger nuclease (ZFN), transcription activator-like effector nuclease (TALEN), and type II clustered regularly interspaced short palindromic repeats (CRISPR)/CRISPR-associated protein 9 (Cas9), is considered a promising approach to correct disease-causing mutations in various human diseases. In particular, hemophilia A can be considered an ideal target for gene modification via engineered nucleases because it is a monogenic disease caused by a mutation in coagulation factor VIII (FVIII), and a mild restoration of FVIII levels in plasma can prevent disease symptoms in patients with severe hemophilia A. In this study, we describe a universal genome correction strategy to restore FVIII expression in induced pluripotent stem cells (iPSCs) derived from a patient with hemophilia A by the human elongation factor 1 alpha (EF1α)-mediated normal *FVIII* gene expression in the *FVIII* locus of the patient. We used the CRISPR/Cas9-mediated homology-directed repair (HDR) system to insert the B-domain deleted from the *FVIII* gene with the human EF1α promoter. After gene targeting, the *FVIII* gene was correctly inserted into iPSC lines at a high frequency (81.81%), and these cell lines retained pluripotency after knock-in and neomycin resistance cassette removal. More importantly, we confirmed that endothelial cells from the gene-corrected iPSCs could generate functionally active FVIII protein from the inserted *FVIII* gene. This is the first demonstration that the *FVIII* locus is a suitable site for integration of the normal *FVIII* gene and can restore FVIII expression by the EF1α promoter in endothelial cells differentiated from the hemophilia A patient-derived gene-corrected iPSCs.

## Hemophilia: One gene therapy fits all

A strategy to restore the expression of the gene encoding blood clotting factor VIII (FVIII) offers new hope to patients with hemophilia A. Hemophilia A is a rare bleeding disorder caused by a variety of mutations in the FVIII gene which affect the function of FVIII protein. At present, the main treatment option relies on the injection of expensive clotting-factor concentrates to restore functional levels of the FVIII. Dong-Wook Kim and colleagues at Yonsei University in Seoul, South Korea, have used genome editing techniques to insert a corrected version of the FVIII gene into stem cells derived from a patient with severe hemophilia A. When these cells differentiated into the cells lining blood vessels they were able to produce and secrete active FVIII protein. This approach offers the attractive possibility of correcting all hemophilia-causing FVIII mutations.

## Introduction

Hemophilia A is a dominant hemophilic disorder, affecting 1 in 5000 males, and is caused by a deficiency in coagulation factor VIII (FVIII)^1,2^. Patients with severe cases of hemophilia A suffer from frequent spontaneous bleeding events in various organs, including the joints and muscles, that can lead to chronic musculoskeletal disabilities^3^. The major treatment for hemophilia A is supplementation with clotting factor, but this requires frequent intravenous infusions (1–3 times in a week for prophylactic treatment) and high costs for clotting factor concentrates^4,5^. Gene therapy of hemophilia A is expected to become a therapeutic alternative to supplementation with clotting factor concentrates. In particular, hemophilia A is a feasible target for gene therapy because increasing the plasma level of FVIII by only 1% causes therapeutic improvements in patients with severe hemophilia A^6,7^.

Recently developed engineered nucleases, including zinc finger nuclease (ZFN), transcription activator-like effector nuclease (TALEN), and type II clustered regularly interspaced short palindromic repeats (CRISPR)/CRISPR-associated protein 9 (Cas9), are already used in gene therapy for various diseases and enable a more sophisticated modification of mutated genes. Moreover, the potential for using engineered nucleases in patient-derived induced pluripotent stem cells (iPSCs) and cell type-specific differentiation techniques provides an unlimited source for future ex vivo cell therapy materials for autologous transplantation^8,9^. Previously, we showed that an inversion genotype can be generated or corrected in human iPSCs using TALEN^10^. We also showed that CRISPR/Cas9 can revert inversion mutations in human iPSC lines derived from patients with intron 1 or intron 22 inversion. We confirmed that transplantation of endothelial cells derived from gene-corrected iPSCs can rescue injury mortality in hemophiliac mice^11^. Others have also used TALEN to insert the exon 23–26 cDNA fragment at the deletion junction of exon 22 and intron 22 in intron 22 inversion patient-derived iPSCs, and found that functionally active FVIII protein was expressed in differentiated cells from gene-corrected iPSC lines^12^. These previous corrections of intron 22 inversion in patient-derived iPSCs are crucial because intron 22 inversion is the most common mutation of hemophilia A, occurring in almost half of patients with severe hemophilia A^13,14^.

However, the other half of severe hemophilia A is caused by various types of mutations, including insertions, deletions, and point mutations^15^. Therefore, to correct all of these mutant types in hemophilia A patients, it is inevitable that large arrays of customized sets of ZFN and TALEN, single-guide RNAs (sgRNAs) for CRISPR/Cas9, and targeting donors will be required. Another possible and universal approach is the insertion of an *FVIII* transgene into a specific site of the genome. This approach is a more likely strategy for dealing with all *FVIII* mutant variants because the *FVIII* transgene can express the functional FVIII protein, regardless of mutant variants of hemophilia A. In this way, a single set of genes targeting an engineered nuclease and the *FVIII* gene donor plasmid is sufficient to address virtually all hemophilia A mutant types.

In this study, we explored the possibility of a universal gene-correction strategy in which the human EF1α promoter-driven *FVIII* gene is expressed in the *FVIII* locus of hemophilia A patient-derived iPSCs by using a CRISPR/Cas9-mediated donor plasmid knock-in. We designed knock-in donor plasmids for an expression cassette with the B-domain deleted form of *FVIII* (BDD-FVIII) and the EF1α promoter for insertion at exon 1 of the *FVIII* locus. Importantly, insertion of the *FVIII* gene resulted in the production of a functionally active FVIII protein from the gene-corrected iPSC line-derived endothelial cells.

## Materials and methods

### Cell cultures

Human embryonic kidney (HEK293) cells were cultured in Dulbecco’s Modified Eagle’s Medium (DMEM) supplemented with 10% (vol/vol) fetal bovine serum (FBS) plus 1% (vol/vol) P/S. FVIII-deleted patient-derived iPSCs (Park, C.Y., 2019, unpublished data), and gene-corrected iPSC lines were maintained on Matrigel (Corning, Corning, NY, USA)-coated cell culture plates in STEMMACS^TM^ iPSC-brew FX (STEMMACS medium; Miltenyi Biotec, Bergisch Gladbach, Germany) medium for feeder-free culture. Briefly, iPSCs were passaged as cells once they reached a confluency of 70–80%. For passaging, we rinsed iPSCs with Dulbecco’s phosphate-buffered saline (dPBS) once and incubated them with Versene solution (Gibco, Grand Island, NY, USA) for 4–5 min. Next, we changed the Versene solution for STEMMACS medium and pipetted cells to dissociate the culture into small clumps. iPSC clumps were split 1:10 and reseeded on a new Matrigel-coated culture dish in STEMMACS medium supplemented with 10 μM of Y27632 (Sigma-Aldrich, St. Louis, MO, USA). The next day, the iPSC culture medium was changed to fresh STEMMACS medium without Y27632, and the medium was refreshed daily.

### sgRNA preparation and validation

We purchased recombinant *Streptococcus pyogenes* Cas9 (SpCas9) and sgRNA expression plasmids from ToolGen (Seoul, Korea). Potential off-target sites that differed by up to three nucleotides from the sgRNA were also provided by ToolGen (Supplementary Table 1). To validate the cleavage activity of the sgRNA, we transfected Cas9 protein and sgRNA expression plasmids into HEK293 cells. Three days after transfection, genomic DNA was purified with DNeasy Blood & Tissue Kits (QIAGEN, Hilden, Germany) and applied to the T7E1 assay as described previously^16^.

### Donor plasmid construction

We used pcDNA4/BDD-FVIII (Addgene #40135) for EF1α-FVIII knock-in donor plasmid construction. First, we introduced a single point mutation in the respective protospacer adjacent motif (PAM) site (C>T, 36 bp downstream from the BDD-FVIII start codon) to evade cleavage by Cas9/sgRNA. Then, the cytomegalovirus (CMV) promoter of the original pcDNA4/BDD-FVIII was substituted with the 1113 bp 5′-homology arm (left arm) cloned from human genomic DNA and inserted into the MfeI/NruI site. The human elongation factor 1 alpha (EF1α) promoter was inserted into the MluI/NruI site between the left arm and the BDD-FVIII open reading frame. We inserted a bovine growth hormone (bGH) polyadenylation signal and the neomycin resistance cassette flanked by *loxP* sites fused by overlapping PCR into the 3′ end of the BDD-FVIII open reading frame using the NotI/MauBI site. Afterward, a 786 bp 3′-homology arm (right arm) was cloned from human genomic DNA and inserted into the PacI/MauBI site. The sequence of the donor plasmid from the 5′ end of the left arm and to the 3′ end of the right arm was confirmed by Sanger sequencing at Cosmogenetech (Seoul, Korea).

### Generation of gene-corrected patient-derived iPSCs

Patient-derived iPSC colonies were pretreated with 10 μM Y27632 for 2 h prior to electroporation. Cells were then washed once with dPBS and dissociated into single cells using TrypLE^TM^ Select (Gibco). iPSC cells (5 × 10^5^) were electroporated with 2 μg Cas9, 2 μg sgRNA expression vector, and 4 μg donor plasmids using a Neon^R^ electroporator (Invitrogen, Carlsbad, CA, USA) as previously described^10^. Transfected cells were plated onto a Matrigel-coated plate with 10 μM Y27632 for 2 days. G418 (100 μg/mL) was added to the culture medium 2 days after electroporation. After 12–14 days of G418 selection, half of the surviving colonies were manually lifted and lysed for genotype as described previously^9^. Correctly targeted colonies were dissociated into single cells and reseeded for expansion and further analysis. To generate single cell-derived correctly targeted iPSCs, we performed an additional three rounds of single colony passaging with G418 selection. After three rounds of single colony passaging and G418 selection, the correctly targeted cell lines underwent excision from the neomycin resistance cassette. We electroporated 2 μg pCAG-Cre:GFP vector (Addgene #13776) into 5 × 10^5^ iPSCs and performed clonal selection without a selection drug.

### PCR analysis of targeted *FVIII* gene knock-in

**Fig. 1:**
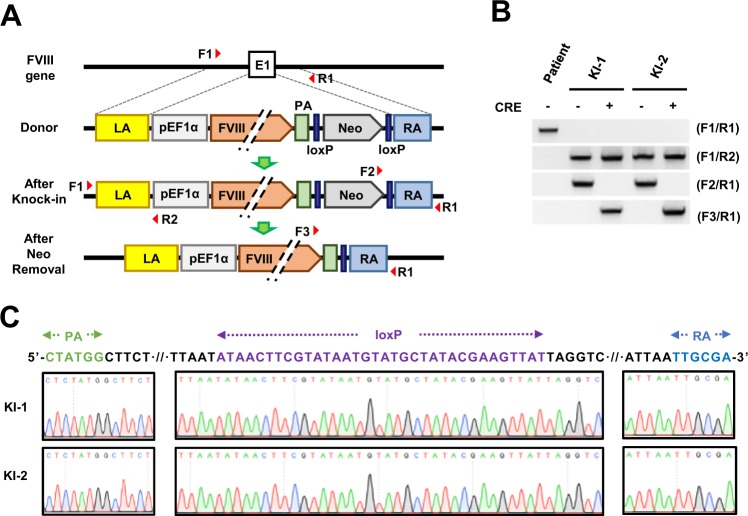
Site-specific integration of the *FVIII* gene at a hemophilia A patient’s *FVIII* locus. **a** A schematic representation of the targeted insertion of the *FVIII* gene at the human *FVIII* locus and the excision of the neomycin resistance cassette. Top depicts exon 1 of the human *FVIII* locus, and below shows donor plasmid, which consists of a 1113 bp left arm (LA), human EF1α promoter (pEF1α), BDD form of *FVIII* (FVIII), bovine growth hormone polyadenylation signal (PA), *loxP*-flanked neomycin resistance cassette (Neo), and 786 bp right arm (RA). The neomycin resistance cassette was removed by *Cre* expression after the knock-in of the donor plasmid. Primers used in PCR analysis are represented by red arrowheads. **b** Genomic PCR analysis of gene-corrected clones before (−) and after (+) *Cre* expression in the gene-corrected cell lines KI-1 and KI-2. The F1/R1 primer pair was used to detect exon 1 (E1) of the *FVIII* locus. The primer sets F1/R2 and F2/R1 were used to detect the knock-in junctions of the 5′ and 3′ ends in correctly targeted clones. The F3/R1 primer pair was used for detecting the removal of the neomycin resistance cassette. Genomic DNA from parental patient iPSCs was used for the control (patient). **c** Partial chromatograms from a 1626 bp PCR amplicon generated with F3/R1, showing the sequences around *loxP* in KI-1 and KI-2 cell lines after removal of the neomycin resistance cassette. Partial bGH poly A signal (PA), *loxP*, and partial right arm (RA) shown in green, purple, and blue, respectively

Genomic DNA was purified using DNeasy Blood & Tissue Kits (QIAGEN) according to the manufacturer’s instructions. We used primer sets specific to the donor plasmid and genomic DNA sequences adjacent to the 5′ and 3′ ends of the integration junction. The target location and sequences are shown in Fig. 1a and Supplementary Table 2. We sequenced PCR amplicons of knock-in junctions at Cosmogentech to verify their identity.

### Analysis of indel frequency

Genomic DNA was isolated from both the patient and corrected iPSC clones using DNeasy Blood & Tissue Kits (QIAGEN). To analyze the indel frequency, we amplified the off-target regions using Phusion polymerase (Thermo Fisher Scientific, MA, USA). The specific primer sets are listed in Supplementary Table 3. Deep-sequencing libraries were generated from the second PCR using the TruSeq HT Dual Index primers. The resulting libraries were subjected to paired-end sequencing using MiSeq (Illumina, San Diego, CA, USA) at LAS, Inc. (Gimpo, Korea) as previously reported^17^.

### In vitro differentiation into three germ layers

We performed the in vitro three-germ layer formation assay as previously described^10,18^. Briefly, iPSC colonies were manually dissected by glass hock and lifted using collagenase type IV (Invitrogen) to generate embryonic bodies (EBs). EBs were cultured on low-attachment cell culture dishes in 5% FBS containing EB culture medium [DMEM/F12 medium containing 4 ng/mL basic fibroblast growth factor (bFGF; PeproTech, Rocky Hill, NJ, USA), 20% knockout serum replacement (Invitrogen), 1% nonessential amino acids (Invitrogen), and 0.1 mM 2-mercaptoethanol (Sigma-Aldrich)]. After 1 week, EBs were plated onto Matrigel-coated dishes and cultured for an additional 10 days for spontaneous differentiation.

### RNA isolation, reverse transcription polymerase chain reaction (RT-PCR), and quantitative PCR (qPCR) analysis

We purified total RNA from patient-derived iPSCs or iPSC-derived endothelial cells with an Easy-Spin Total RNA Extraction Kit (Intron Biotechnology, Seongnam, Korea) according to the manufacturer’s instructions. Then, we used 1 μg purified total RNA to generate cDNA with PrimeScript^TM^ RT Master Mix (TAKARA BIO, Inc., Otsu, Japan) and performed qPCR using SYBR^®^ Premix ExTaq^TM^ (TAKARA BIO, Inc.). mRNA levels were quantified using the CFX96 Real-Time System (Bio-Rad, Hercules, CA, USA). Ct values of GAPDH were used as an endogenous reference to normalize the relative expression levels of target genes based on their Ct values. For semiquantitative RT-PCR, we used EmeraldAmp^®^ GT PCR Master Mix (TAKARA BIO, Inc.) to amplify the target site according to the manufacturer’s instructions. Primer sequences used for RT-PCR or qPCR are shown in Supplementary Table 2.

### Differentiation of endothelial cells from iPSCs

We performed endothelial cell differentiation from iPSCs using a previously described protocol with minor modifications^19^. Briefly, iPSCs were dissociated with Versene solution and transferred to a new Matrigel-coated dish in STEMMACS medium supplemented with 10 μM Y27632. On day 0 of differentiation, iPSCs were treated with 6 μM CHIR99021 (Tocris Bioscience, Bristol, UK) in STEMdiff^TM^ APEL^TM^2 medium (STEMCELL technologies, Vancouver, BC, Canada) for 2 days. On day 2, CHIR99021-containing medium was changed to STEMdiff^TM^ APEL^TM^2 medium with 25 ng/mL BMP4 (ProSpec, NJ, USA), 10 ng/mL bFGF (PeproTech), and 50 ng/mL VEGF-A (PeproTech) for 2 days. On day 4, cells were detached with TrypLE^TM^ select, transferred to new culture dishes and cultured in endothelial cell growth medium-MV2 (ECGM-MV2; Promocell, Heidelberg, Germany) supplemented with 50 ng/mL VEGF-A. The ECGM-MV2 with VEGF-A was refreshed every 2 days. On day 8 of differentiation, the resulting endothelial cells were applied to the appropriate assays.

### Immunocytochemistry

For immunofluorescent staining, we fixed cells on glass slides with a 4% paraformaldehyde solution for 10 min, washed three times with PBS, and permeabilized with PBS containing 0.1% Triton X-100 for 10 min at room temperature. After blocking in blocking buffer (PBS containing 2% bovine serum albumin) for 1 h at room temperature, the samples were incubated with primary antibody diluted in blocking buffer at 4 °C overnight. The following primary antibodies were used: rabbit anti-OCT4 (1:200, Santa Cruz Biotechnology, Dallas, TX, USA), mouse anti-SSEA4 (1:200, Millipore, Billerica, MA, USA), rabbit anti-NESTIN (1:1000, Millipore), goat anti-SOX17 (1:200, Santa Cruz Biotechnology), mouse anti-α-SMA (1:400, Sigma-Aldrich), mouse anti-CD31 (1:200, BD Biosciences, San Jose, CA, USA), and rabbit anti-vWF (1:500, Millipore). After washing three times with PBS, we incubated samples with fluorescence-tagged secondary antibodies (Alexa Fluor^®^ 488 or Alexa Fluor^®^ 594, 1:1000, Invitrogen) in PBS for 30 min at room temperature. Samples were washed again three times with PBS and mounted onto slides using 4′,6-diamidino-2-phenylindole-containing mounting medium (Vector Laboratories, Burlingame, CA, USA). All images were captured with a fluorescence microscope (Eclipse Ti-U, Nikon Instruments Inc., Tokyo, Japan).

### FVIII activity assay

On day 8 of differentiation, we changed the endothelial cell culture medium to phenol red free ECGM-MV2 medium with 50 ng VEGF-A. After 24 h incubation, the supernatants were collected and concentrated 20 times using centrifugal filter units (Millipore). FVIII activity in the concentrated culture supernatant was measured using the Coamatic^®^ Factor VIII Chromogenic Assay Kit (Instrumentation Laboratory, Bedford, MA, USA) according to the manufacturer’s instructions.

### Statistics

All data values are expressed as the mean ± standard error of the mean (S.E.M.) unless otherwise indicated. Statistical significance was estimated using Student’s *t*-test. A resulting *p*-value < 0.01 was considered statistically significant.

## Results

### Sequence analysis of the *FVIII* gene breakpoint from a patient with severe hemophilia A

In this study, we used an iPSC line derived from a hemophilia A patient with a gross deletion (exon 8–exon 22) at the *FVIII* locus. Targeted genotype PCR from intron 7 to intron 22 and Sanger sequencing analysis revealed a gross deletion of 94,172 bp from exon 8 to intron 22 at the patient’s *FVIII* locus (Supplementary Fig. 1a). We identified the mRNA sequences around the deletion junction in the patient’s *FVIII* locus by using mRNA transcripts from the patient-derived iPSC line. RT-PCR and Sanger sequencing analysis targeting exon 7–exon 23 showed that partial exon 8 and intron 22 sequences were spliced out and that exons 7 and 23 were directly linked to make a shorter version of the *FVIII* mRNA, which also generated a premature stop codon in exon 23 (Supplementary Fig. 1b).

### A strategy for the restoration of FVIII expression based on donor plasmids knock-in in the *FVIII* locus

We hypothesized that insertion of the human EF1α promoter-driven *FVIII* gene in exon 1 of the *FVIII* locus would express functionally active FVIII protein regardless of the mutant type. Therefore, we designed a nuclease targeting 34 bp downstream from the start codon in exon 1 of the human *FVIII* locus on chromosome X for homology-directed repair (HDR)-mediated knock-in (Supplementary Fig. 2a). Next, we tested the cleavage efficiency of the Cas9/sgRNA by transient expression of the sgRNA and Cas9 expression vector in HEK293 cells. A subsequent T7E1 analysis and Sanger sequencing of the sgRNA target site revealed that the Cas9/sgRNA induced various indels at the target site with a frequency of 11% (Supplementary Fig. 2b, c).

Next, we designed a donor plasmid to restore FVIII expression using a BDD-FVIII cDNA. The donor plasmid was designed to use the human EF1α promoter for BDD-FVIII expression. Based on this concept, the donor plasmid included the EF1α promoter, the BDD-FVIII cDNA, a bGH polyadenylation signal, a *loxP*-flanked neomycin resistance cassette, and the left and right arms (Fig. 1a).

We then introduced the CRISPR/Cas9 and sgRNA expression vectors, and the donor plasmid into hemophilia A patient-derived iPSCs to create EF1α-BDD-FVIII knock-in iPSC lines. After drug selection with G418, genomic DNA of the surviving colonies was collected for initial PCR screening to identify correctly targeted colonies by amplifying each 5′ and 3′ knock-in junction with the specific primer set of F1/R2 and F2/R1 (Fig. 1a). PCR screening results indicated that the donor plasmid inserted into exon 1 of the *FVIII* locus at a frequency of 81.81% (18 colonies positive from a total of 22 colonies) (Supplementary Fig. 3a, b). Then, we obtained two clones (KI-1 and KI-2) after an additional three rounds of single colony expansion and G418 selection. Targeted PCR analysis of 5′ and 3′ knock-in junctions and Sanger sequencing analysis of PCR amplicons showed that donor plasmids were correctly targeted in exon 1 of the patient’s *FVIII* locus (Fig. 1b, Supplementary Fig. 4a, b). Then, KI-1 and KI-2 cell lines were subjected to removal of the neomycin resistance cassette by *Cre* recombinase expression. Targeted genomic DNA PCR using a specific primer set (F3/R1, Fig. 1a) and Sanger sequencing of the amplified PCR amplicons confirmed the complete removal of the neomycin resistance cassette in the knock-in cell line after *Cre* expression and single colony expansion (Fig. 1b, c).

### Pluripotency and off-target analysis of gene-corrected patient-derived iPSCs

**Fig. 2:**
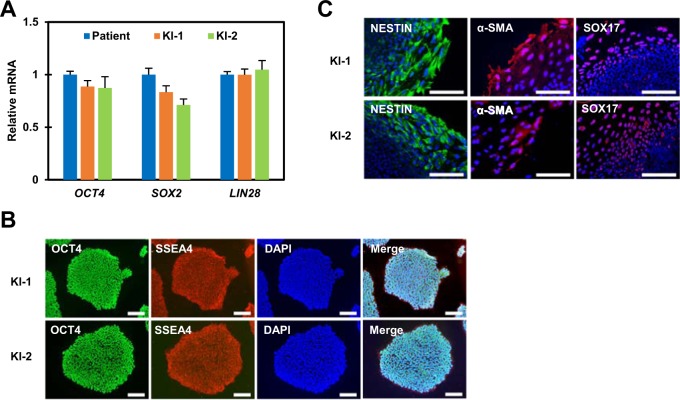
Pluripotency analysis of gene-corrected iPSC lines. **a** Quantitative real-time PCR (qPCR) analysis of *OCT4*, *SOX2*, and *LIN28* in parental patient cells and gene-corrected iPSC lines. *GAPDH* was used to normalize gene expression. **b** Immunofluorescence staining to indicate expression of the pluripotency markers OCT4 (green) and SSEA4 (red) of gene-corrected iPSC clones. Nuclei were labeled with 4′,6-diamidino-2-phenylindole (DAPI; blue) (scale bar, 200 μm). **c** Immunofluorescence staining shows the expression of marker proteins, representing the ectoderm (NESTIN, green), the mesoderm (α-SMA, red), and the endotherm (SOX17, red). Nuclei were labeled with DAPI (blue) (scale bar, 200 μm)

We determined whether the KI-1 and KI-2 gene-corrected cell lines remained pluripotent after gene targeting. Our quantitative real-time PCR (qPCR) results showed that gene-corrected cell lines expressed the pluripotent marker genes *OCT4*, *SOX2*, and *LIN28* at levels similar to those of the parental hemophilia A patient-derived iPSC line (Fig. 2a). We also confirmed uniform expression of OCT4 and SSEA4 in iPSC colonies by immunocytochemistry analysis (Fig. 2b). In vitro three germ layer formation assays showed that these lines could be differentiated into three germ layers (Fig. 2c). We then sequenced off-target sites of the sgRNA in the gene-corrected iPSC clone KI-1. We obtained a list of potential off-target sites from ToolGen (Seoul, Korea) that differed from the on-target site by up to three nucleotides. We selected four potential off-target sites from the list and subjected these sites to targeted deep-sequencing. No significant mutations were found in any of the analyzed off-target sites in the corrected KI-1 cell line (Supplementary Fig. 5).

### Restoration of FVIII expression in the gene-corrected iPSC-derived endothelial cells

**Fig. 3:**
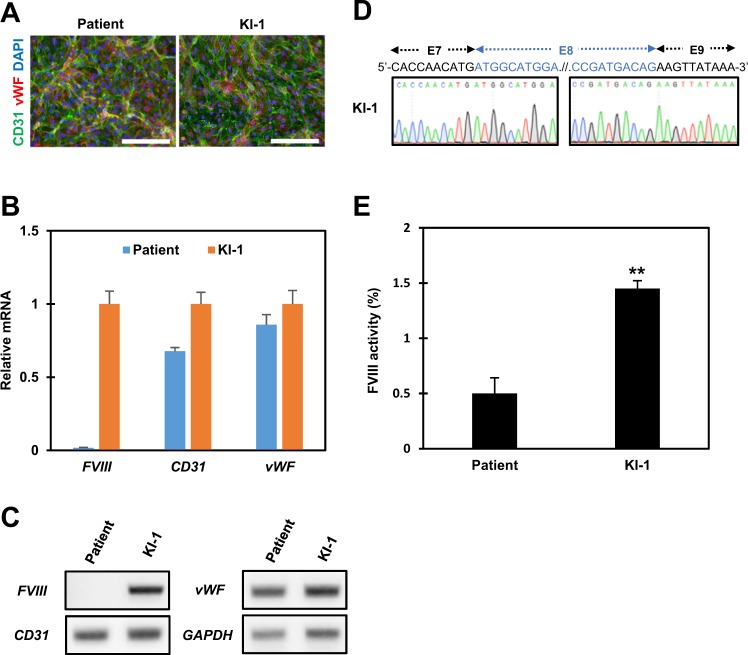
Restoration of FVIII expression in the gene-corrected iPSC-derived endothelial cells. **a** Immunofluorescence staining of endothelial cell markers CD31 (green) and vWF (red) differentiated from the parental patient and gene-corrected iPSC clones. Nuclei were labeled with DAPI (blue) (scale bar, 200 μm). **b** qPCR analysis of *FVIII*, *CD31*, and *vWF* in cells from the patient and from gene-corrected iPSC line-derived endothelial cells. The *FVIII* transcript was amplified with primers based on exon 7 and exon 10. **c** Expression of *FVIII* and endothelial cell markers *CD31* and *vWF* were analyzed by RT-PCR. Endothelial cells were derived from cells from the patient and gene-corrected iPSC lines. **d** Sanger sequencing analysis of *FVIII* amplicons from gene-corrected iPSC-derived endothelial cells with restored exon 7–exon 9 sequence. **e** The FVIII activity of cell culture supernatants from either patient-corrected or gene-corrected iPSC-derived endothelial cells. FVIII activity was determined in 5 × 10^5^ endothelial cells per single detection. **p < 0.01 compared to the patient control

We then asked whether endothelial cells from gene-corrected iPSC lines could restore FVIII expression. We differentiated the gene-corrected KI-1 cell line into endothelial cells^19^ and then examined the expression of *FVIII* mRNA and the secretion of functionally active FVIII protein. After 8 days of differentiation, the endothelial nature of cells was confirmed by immunocytochemistry and PCR analysis. Differentiated cells were positive for staining of the endothelial cell markers CD31 and vWF (Fig. 3a). Then, we used PCR analysis to evaluate the expression of *FVIII* and the endothelial cell markers *CD31* and *vWF* in iPSC-derived endothelial cells. We used the primer set targeting exon 7–exon 10 to discriminate between the patient’s *FVIII* and knocked-in *BDD-FVIII* mRNA. Our qPCR and RT-PCR results showed no significant differences in the endothelial cell markers *CD31* and *vWF* between parental patient iPSCs and the gene-corrected KI-1 cell line (Fig. 3b, c). However, as we expected, the *FVIII* transcript was only detected in the KI-1 cell line-derived endothelial cells, as shown by both qPCR and RT-PCR analyses (Fig. 3b, c). We also confirmed by Sanger sequencing that the PCR amplicon had normal exon 7–exon 9 sequences of *FVIII* cDNA (Fig. 3d).

Finally, we performed the FVIII activity assay to identify whether functionally active FVIII protein was secreted from gene-corrected iPSC-derived endothelial cells. We confirmed a significant increase in FVIII activity (2.9-fold increase) compared to the control in the endothelial cell culture supernatant (Fig. 3e). Altogether, our data showed that the insertion of the normal *FVIII* gene into exon 1 of the mutant *FVIII* locus can generate a functional FVIII protein in iPSC-derived endothelial cells.

## Discussion

In this study, we used iPSCs derived from a severe hemophilia A patient with a gross deletion of *FVIII* from exon 8 to exon 22. With this mutation, one possible approach for restoring FVIII expression might be achieved by inserting the cDNA sequence spanning exon 8–exon 22 in the patient’s *FVIII* locus. However, this approach only applies to one specific event but not for other hemophilia A *FVIII* mutant variants. As we discussed above, we hypothesized that insertion of the *FVIII* transgene into a specific locus of the human genome is a suitable method for universal gene correction to overcome this limitation. In the case of hemophilia B, the second most abundant hemophilia type caused by a mutation of factor IX (FIX), there have been efforts to use the *FIX* locus itself to express the *FIX* gene. It is known that insertion of the *FIX* exon 2 to exon 8 sequence in intron 1 of the human *FIX* gene in a humanized hemophilia B mouse model restored FIX expression via ZFN-mediated in vivo gene correction^20^. Another recent report also showed that insertion of the *FIX* cDNA at exon 1 of the human *FIX* locus restored FIX expression in gene-corrected hemophilia B patient iPSC-derived hepatocytes, both in in vitro and in vivo models^21^.

Similar to these approaches, we designed a universal strategy to restore FVIII expression in patient-derived iPSCs. We inserted a human *FVIII* gene with EF1α promoter-driven expression into exon 1 of the *FVIII* locus in hemophilia A patient-derived iPSCs with high efficiency (81.81% in initial screening). We also checked the indel frequencies at off-target sites because unwanted mutations at off-target sites are an important risk factor for using engineered nucleases^22,23^. Our targeted deep sequencing data suggest that there were no significant mutations in the analyzed off-target sites.

We confirmed the expression of the *FVIII* transcript and of the functionally active FVIII protein from gene-corrected iPSC-derived endothelial cells. Recent findings indicate that liver sinusoidal endothelial cells are a major source of FVIII production; however, other endothelial cell types, such as microvascular and lymphatic endothelial cells, can also generate the FVIII protein^24–26^. Our study and other previous studies also showed that endothelial progenitor cells from human iPSCs could express *FVIII* mRNA and functionally active FVIII protein^11,12^. Moreover, FVIII-transduced human primary endothelial cell progenitor cells are widely used for research into ex vivo therapy for hemophilia A^27–29^.

Our results show that the insertion of the B-domain deleted form of the *FVIII* gene with an EF1α promoter restored FVIII expression in gene-corrected iPSC-derived endothelial cells. We used the BDD-FVIII because it is known that the B-domain is unnecessary for the coagulation activity of FVIII. Moreover, BDD-FVIII has a relatively small size (4.3 kb compared to the 7 kb full-length *FVIII* cDNA), and an enhanced expression capability compared to the full-length *FVIII* cDNA, so BDD-FVIII is widely used in gene therapy for hemophilia A^30–32^. However, it is also known that both B-domain deleted and full-length *FVIII* cDNA have transcriptional repressor sequences that cause inefficient transcription^33,34^. Additionally, deletion of the B-domain also results in a reduced rate of FVIII secretion because it is related to the normal protein folding and efficient secretion of FVIII^35^. In particular, a significant portion of the primary translated BDD-FVIII protein is misfolded and ultimately degraded^36^. Moreover, the half-life of BDD-FVIII is shorter by ~3 h compared with normal FVIII (~12 h)^37^. These properties of BDD-FVIII might have mildly increased FVIII activity (2.9-fold increase) in our gene-corrected iPSC-derived endothelial cells, even though we used the EF1α promoter for enhanced FVIII expression of BDD-FVIII at the human locus. Moreover, because the human *FVIII* locus is located on the X chromosome, only one copy of EF1α-driven *FVIII* mRNA transcription occurs per gene-corrected iPSC-derived endothelial cell. Therefore, we found relatively low expression of FVIII compared to the viral transduction of *FVIII* in human primary cells and can result in multiple *FVIII* transgene insertions in one cell.

We hypothesize that using a modified coding sequence of *FVIII* with enhanced transcriptional and secretion abilities might address these limitations in our future approach. Previous reports found that the insertion of intron 1 of the *FIX* gene into human *FVIII* cDNA or a hybrid of porcine *FVIII* and human *FVIII* cDNA enhanced the production or coagulant activity of FVIII^38,39^. Introducing 226 amino acids with an N-glycosylation site to the BDD form of FVIII also yielded a 10-fold increase in FVIII secretion^40^. Codon-optimized *FVIII* resulted in a 29–44-fold enhancement of FVIII expression, and delivery of codon-optimized *FVIII* via a lentiviral vector resulted in FVIII levels in hemophilic mice that were more than 200% of those found in a normal human^41^. Although we could not use an improved version of FVIII in our experiment, the findings may provide enhanced FVIII expression and secretion abilities for future studies using our gene correction system.

In this research, we provided evidence that insertion of the *FVIII* gene with an EF1α promoter at the *FVIII* locus could restore FVIII expression in endothelial cells from hemophilia A patient-derived iPSCs. Although we used only one patient-derived iPSC line in this study, our gene correction strategy is applicable to a broad spectrum of *FVIII* mutations in hemophilia A, because the *FVIII* gene inserted at the patient’s *FVIII* locus is expressed regardless of *FVIII* mutant variation. These first proof-of-concept experiments demonstrate that the insertion of the EF1α promoter with the *FVIII* gene in the human *FVIII* locus is a suitable strategy for the restoration of FVIII expression, and provides a valuable and universal tool for future ex vivo cell therapy for patients with hemophilia A.

## Supplementary information

Supplementary Information
