# Supplementary material for: Restoration of FVIII expression by targeted gene insertion in the FVIII locus in hemophilia A patient-derived iPSCs
Source: Exp Mol Med. 2019 Apr 17;51(4):45. doi: 10.1038/s12276-019-0243-1 (PMC6470126; doi:10.1038/s12276-019-0243-1)
Supplement: Supplementary file 1 — Supplementary Information [file 12276_2019_243_MOESM1_ESM.docx]

**Restoration of FVIII expression by targeted gene insertion in the *FVIII* locus in hemophilia A patient-derived iPSCs**

**Jin Jea Sung^1,2^, Chul-Yong Park^1,3^,** [**Joong Woo Leem**](https://www.ncbi.nlm.nih.gov/pubmed/?term=Leem%20JW%5BAuthor%5D&cauthor=true&cauthor_uid=28751784)**^1^, Myung Soo Cho^4^, and Dong-Wook Kim^1,2,3^**

**^1^Department of Physiology, Yonsei University College of Medicine, 50-1 Yonsei-ro Seodaemun-gu, Seoul 03722, Korea**

**^2^Brain Korea 21 PLUS Program for Medical Science, Yonsei University College of Medicine, 50-1 Yonsei-ro, Seodaemun-gu, Seoul 03722, Korea**

**^3^Severance Biomedical Research Institute, Yonsei University College of Medicine, 50-1 Yonsei-ro Seodaemun-gu, Seoul 03722, Korea**

**^4^S. Biomedics Co., Ltd., Seoul, South Korea**

**These authors contributed equally: Jin Jea Sung, Chul-Yong Park**

**Correspondence: D-W. Kim (dwkim2@yuhs.ac)**


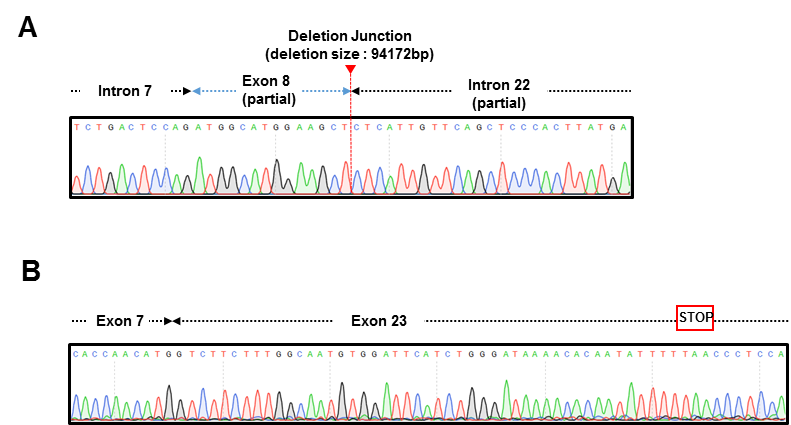


**Supplementary Figure 1. Breakpoint analysis of the *FVIII* locus in a hemophilia A patient with a gross deletion.** **a** Sanger sequencing analysis of deletion junction between exon 8 and intron 22. The deletion junction is indicated by the red arrowhead. **b** Sanger sequencing analysis showing RT-PCR amplicons of the exon 7 and exon 23 junction in *FVIII* transcripts from the hemophilia A patient. Premature stop codon caused by frameshift shown by red box.


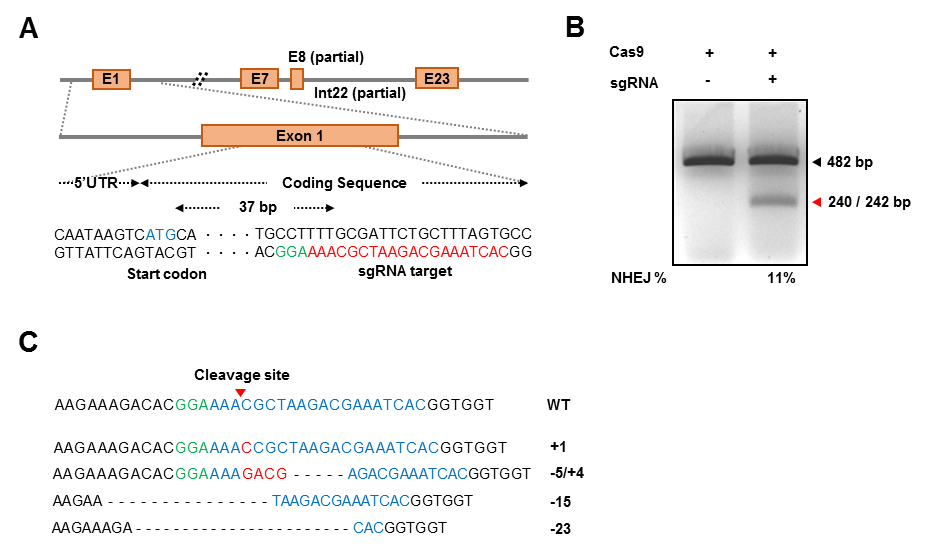


**Supplementary Figure 2. sgRNA design and validation of nuclease activity. a** Schematic overview showing the sgRNA target site in exon 1 of patient’s *FVIII* locus. Top line depicts structure of the *FVIII* locus with the gross deletion of exon 8 to exon 22. The DNA sequences shown include a partial 5′ untranslated region (5′ UTR), start codon and sgRNA target site 37 bp downstream from the start codon in exon 1 of the *FVIII* locus. The start codon is shown in blue, the protospacer adjacent motif (PAM), and sgRNA target are shown in green and red. **b** Non-homologous end joining (NHEJ) frequency with the T7E1 assay. T7E1-treated PCR products amplified from genomic DNA of HEK293 cells transfected with Cas9 and sgRNA expression vector. The black arrowhead indicates on-target 482 bp PCR amplicon. The red arrowhead indicates 240 and 242 bp fragments cleaved by T7E1. **c** Indel patterns induced by the targeting of Cas9/sgRNA in HEK293 cells was analyzed by PCR amplification and Sanger sequencing. The number of bases generated by insertion (red) or removed by deletion (-) are shown on the right. The red arrowhead indicates a cleavage site. The PAM and target sequences are shown in green and blue.


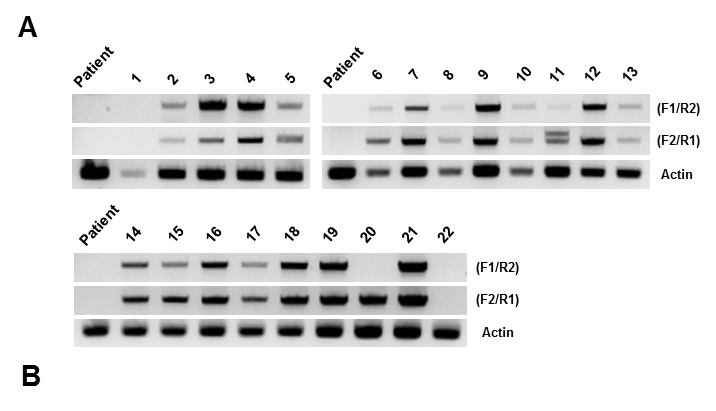


| **Clones**  **picked** | **Targeted**  **integrations** | **Targeting**  **efficiency (%)** |
| --- | --- | --- |
| 22 | 18 | 81.81% |

**Supplementary Figure 3. The primary PCR screening of the survived clones after drug selection. a** Genomic DNA of survived colonies (1-22) were applied to PCR screening to evaluate the site-specific integration of the *FVIII* transgene in the *FVIII* locus by using primer sets F1/R2 (5’ knock-in junction), F2/R1 (3’ Knock-in junction) shown in Fig 1a, b. The predicted size of PCR products are 1643 bp for F1/R2, 1324 bp for F2/R1. 295 bp β-actin bands were used as internal reference. Genomic DNA from parental patient iPSCs was used for the control (Patient). **b** Summary of knock-in efficiency based on the primary PCR screening.


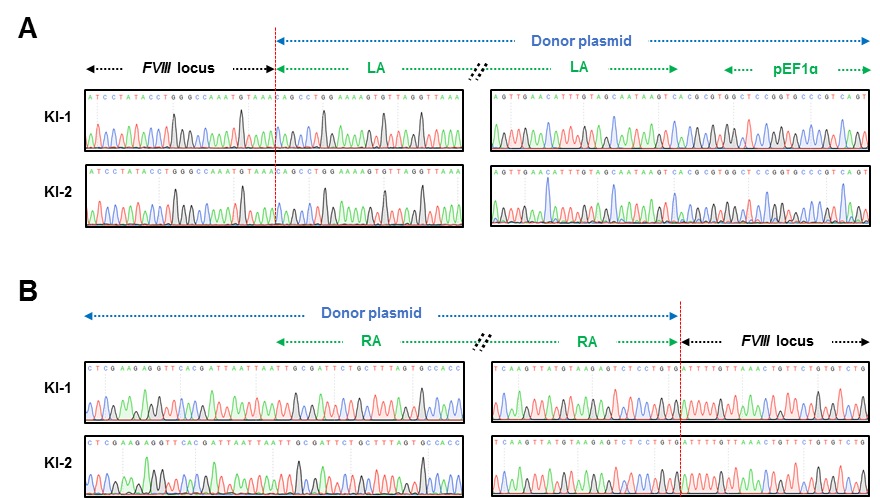


**Supplementary Figure 4. Sequence analysis of *FVIII* gene knock-in iPSCs related to Fig. 1.** PCR amplicons of the integration junction were analyzed by Sanger sequencing. **a** Partial chromatograms from 1643 bp PCR amplicons were generated with the F1/R2 primer set shown in Fig 1a, b. Sequences in chromatograms represent sequences at the integration junction between the left arm and exon 1 of the *FVIII* locus. **b** Partial chromatograms showing sequences at the integration junction between the right arm and exon 1 of the *FVIII* locus. We generated 1324 bp PCR amplicons with the F2/R1 primer set shown in Fig 1a, b. DNA sequence from the *FVIII* locus or donor plasmid is indicated by a black or blue dotted line with arrowhead. DNA sequence from left arm (LA), right arm (RA), and EF1α promoter (pEF1α) is also shown by a green dotted line with arrowhead. The border between homology arms and endogenous DNA sequence of the *FVIII* locus is indicated by red dotted lines.


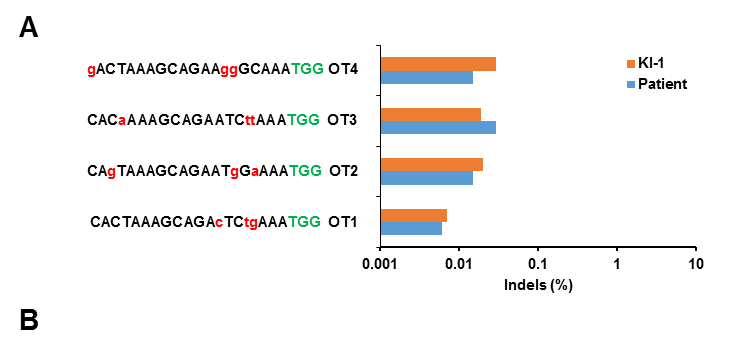


| **Patient** | **KI-1** |
| --- | --- |
| 39727 | 41966 |
| 30983 | 36549 |
| 32458 | 30637 |
| 31277 | 43207 |

**Supplementary Figure 5. Analysis of off-target sites in the gene-corrected iPSC line using targeted deep sequencing. a** We analyzed four off-target sites among 16 total off-target sites that differed by up to three nucleotides from the targeted site in the gene-corrected iPSC line KI-1. Mismatched nucleotides and PAM sequences are shown in red and green. b The number of total reads are presented.

**Supplementary Table 1.** **List of off-targets**

| **Off targets** | **Target site** | **location** | **directions** |
| --- | --- | --- | --- |
| OT1 | CACTAAAGCAGAcTCtgAAATGG | Chr8 | - |
| OT2 | CAgTAAAGCAGAATgGaAAATGG | Chr11 | - |
| OT3 | CACaAAAGCAGAATCttAAATGG | Chr5 | + |
| OT4 | gACTAAAGCAGAAggGCAAATGG | Chr10 | - |
| OT5 | aAtTAAAGCAGAATCtCAAACAG | Chr2 | - |
| OT6 | CACTAAAGgAGAAatGCAAACAG | Chr2 | - |
| OT7 | CACTAAAGCAaAATaaCAAATAG | Chr5 | + |
| OT8 | CAaTAAAGCAGAAaCaCAAAAAG | Chr5 | - |
| OT9 | CACTggAGCAGAATaGCAAAGGG | Chr6 | + |
| OT10 | CAtTAAAGCAaAATtGCAAATGG | Chr7 | - |
| OT11 | tACTAAAtCAGAATCtCAAAGGG | Chr8 | - |
| OT12 | CACTtAAGCAaAATgGCAAAGGG | Chr9 | + |
| OT13 | CcCTAAAGCAGcATCGCAgACAG | Chr10 | + |
| OT14 | CACgAAAGaAGAATCtCAAAAGG | Chr11 | + |
| OT15 | CctTAAAGCAGAgTCGCAAAGAG | Chr12 | - |
| OT16 | aACTAcAGCAGAATaGCAAAGGG | ChrX | - |

**Supplementary Table 2**. **List of primers for PCR analysis**

| **Primers** | **Sequence** | **Targets** |
| --- | --- | --- |
| F8-Int7-F | TGATTTGTGGCAAGAAGGGA | Sanger sequencing for deletion junction in FVIII deletion patient |
| F8-Int22-R | CCACACAGTTACAACCATATG |  |
| F8-RT-E6-F1 | GCCTGGCCTAAAATGCACAC | Sanger sequencing for exon7 – exon 23 junction in patient’s FVIII cDNA |
| F8-RT-E23-R1 | ATGAGTTGGGTGCAAACGGA |  |
| F1 | CTGTCATCTCTGCATCCTTGTACC | Screening knock-in clones and sanger sequencing |
| R1 | GGGAGCCAAACAGAAAGAACC |  |
| F2 | TCTATGGCTTCTGAGGCGGA |  |
| R2 | CCGTTGCGAAAAAGAACGTTCAC |  |
| F3 | ACAGGACCTCTACTGAGCGG |  |
| β-actin | TCACCCACACTGTGCCCATCTACGA |  |
| β-actin | CAGCGGAACCGCTCATTGCCAATGG |  |
| F8-T7E1-F | TAAAAAGGAAGCAATCCTATCGG | On-target PCR for T7EI assay |
| F8-T7E1-R | TGCACACCTTACCCAGAAATG |  |
| GAPDH-F | TGCACCACCAACTGCTTAGC | qRT-PCR for pluripotency  markers |
| GAPDH-R | GGCATGGACTGTGGTCATGAG |  |
| OCT4-F | CCTCACTTCACTGCACTGTA |  |
| OCT4-R | CAGGTTTTCTTTCCCTAGCT |  |
| SOX2-F | TTCACATGTCCCAGCACTACCAGA |  |
| SOX2-R | TCACATGTGTGAGAGGGGCAGTGT |  |
| LIN28-F | AGCCATATGGTAGCCTCATGTCCG |  |
| LIN28-R | TCAATTCTGTGCCTCCGGGAGCAG |  |
| CD31-F | TGCGAATCGATCAGTGGA | qRT-PCR for Endothelial cell  markers |
| CD31-R | ACCGGGGCTATCACCTTC |  |
| vWF-F | TCGGGCTTCACTTACGTTCT |  |
| vWF-R | CCTTCACTCGGACACACTCA |  |
| F8-RT-E7–F | TCT TGT GAG GAA CCA TCG CC | qRT-PCR for F8 |
| F8-RT-E10–R | ACA TCA GTG ATT CCG TGA GGG |  |

| **Primers** | **Sequence** |
| --- | --- |
| FVIII-OT1-F | TCGTCGGCAGCGTCAGATGTGTATAAGAGACAGGCAAGTACCTTGTATTAGAACT |
| FVIII -OT1-R | GTCTCGTGGGCTCGGAGATGTGTATAAGAGACAGCTGACCAAAGCCTTTTTCATC |
| FVIII -OT2-F | TCGTCGGCAGCGTCAGATGTGTATAAGAGACAGCCACTTGAAAAATCAAAGTTCAAG |
| FVIII -OT2-R | GTCTCGTGGGCTCGGAGATGTGTATAAGAGACAGGTGCCTTAGTAGATTAACAGCT |
| FVIII -OT3-F | TCGTCGGCAGCGTCAGATGTGTATAAGAGACAGCCTAATTTAGAGAAAGAGTAAAGGC |
| FVIII -OT3-R | GTCTCGTGGGCTCGGAGATGTGTATAAGAGACAGCTAAGGCTAGCTGTTAGTTCAC |
| FVIII -OT4-F | TCGTCGGCAGCGTCAGATGTGTATAAGAGACAGCTCTGGTAAACACTCTTTCTAC |
| FVIII -OT4-R | GTCTCGTGGGCTCGGAGATGTGTATAAGAGACAGCTAAACCCAGCATGTAGTTCTA |

**Supplementary Table 3. List of primers for targeted deep sequencing**
